# On the Use of Markov Models in Pharmacoeconomics: Pros and Cons and Implications for Policy Makers

**DOI:** 10.3389/fpubh.2020.569500

**Published:** 2020-10-30

**Authors:** Andrea Carta, Claudio Conversano

**Affiliations:** Department of Business and Economics, University of Cagliari, Cagliari, Italy

**Keywords:** pharmacoeconomics, cost-utility analysis, Markov models, Dengue fever, incremental cost-effectiveness ratio, willingness to pay

## Abstract

We present an overview of the main methodological features and the goals of pharmacoeconomic models that are classified in three major categories: regression models, decision trees, and Markov models. In particular, we focus on Markov models and define a semi-Markov model on the cost utility of a vaccine for Dengue fever discussing the key components of the model and the interpretation of its results. Next, we identify some criticalities of the decision rule arising from a possible incorrect interpretation of the model outcomes. Specifically, we focus on the difference between median and mean ICER and on handling the willingness-to-pay thresholds. We also show that the life span of the model and an incorrect hypothesis specification can lead to very different outcomes. Finally, we analyse the limit of Markov model when a large number of states is considered and focus on the implementation of tools that can bypass the lack of memory condition of Markov models. We conclude that decision makers should interpret the results of these models with extreme caution before deciding to fund any health care policy and give some recommendations about the appropriate use of these models.

## 1. Introduction

In the year 2016 US and EU spent 17.3 and 9.9% of their GDP in healthcare, respectively (1). Therefore, health expenditure is one of the largest items in their national budget and adequate spending and allocation of budget resources to healthcare is an open problem worldwide. To attenuate risks linked to this resource allocation problem, Pharmacoeconomics has recently gained more and more consideration. It refers to the branch of health economics, which compares and analyses the value of one health policy to another. All the pharmacoeconomic analyses estimate the cost of an intervention strategy or of a new pharmaceutical product. They include health consequences expressed in terms of monetary value, efficacy or enhanced quality of life. Perspectives for pharmacoeconomic studies include institutional, provider, patient, governmental and societal. Identifying the right perspective is a fundamental task. A different perspective may alter significantly the cost benefit analysis (2).

Pharmacoeconomic evaluations essentially differ based on the category of outcomes used. A classification of methods used in pharmacoeconomics distinguishes among three major categories (2, 3): cost-benefit analysis (CBA), cost-effectiveness analysis (CEA), and cost-utility analysis (CUA). In CBA, both costs and benefits of a new drug or of a health intervention are put in monetary terms, although it is difficult to attach a monetary value to the health outcomes. In CEA, two or more alternative strategies are compared by measuring the costs and outcomes of each; outcomes can be assessed as natural units (e.g., life-year gained, reduced mortality, morbidity, blood pressure etc.). In CUA, the intervention outcomes are measured in terms of utility or preference; these outcomes are usually expressed in terms of disability-adjusted life years (DALYs) or in quality-adjusted life years (QALYs). One DALY can be seen as one lost year of healthy life. DALYs are computed as the sum of Years Lost due to Disability (YLD) for people living in hill-health condition and the Years of Life Lost (YLL) due to premature mortality. QALY, instead, is measured on a scale from zero (representing death) to one (representing one year of perfect health) and it is routinely used to measure the outcome of health evaluations for all kind of individuals and for all kind of drugs or diseases. Thus, it can be optimally implemented for comparisons across programs. When a CEA or a CUA is accomplished, the incremental cost effectiveness ratio (ICER) is used as a decision rule. ICER tell us how much money invested in the new drug or in the new therapy is necessary to gain one unit of effect, such as life-year gained or one unity of utility, such as QALY. For two alternative policies, say A and B, ICER is computed as

(1) $$\begin{array}{l} ICER\left(A,B\right)=\frac{cost_{A}-cost_{B}}{Effect_{A}-Effect_{B}} \end{array}$$

**Figure 1:**
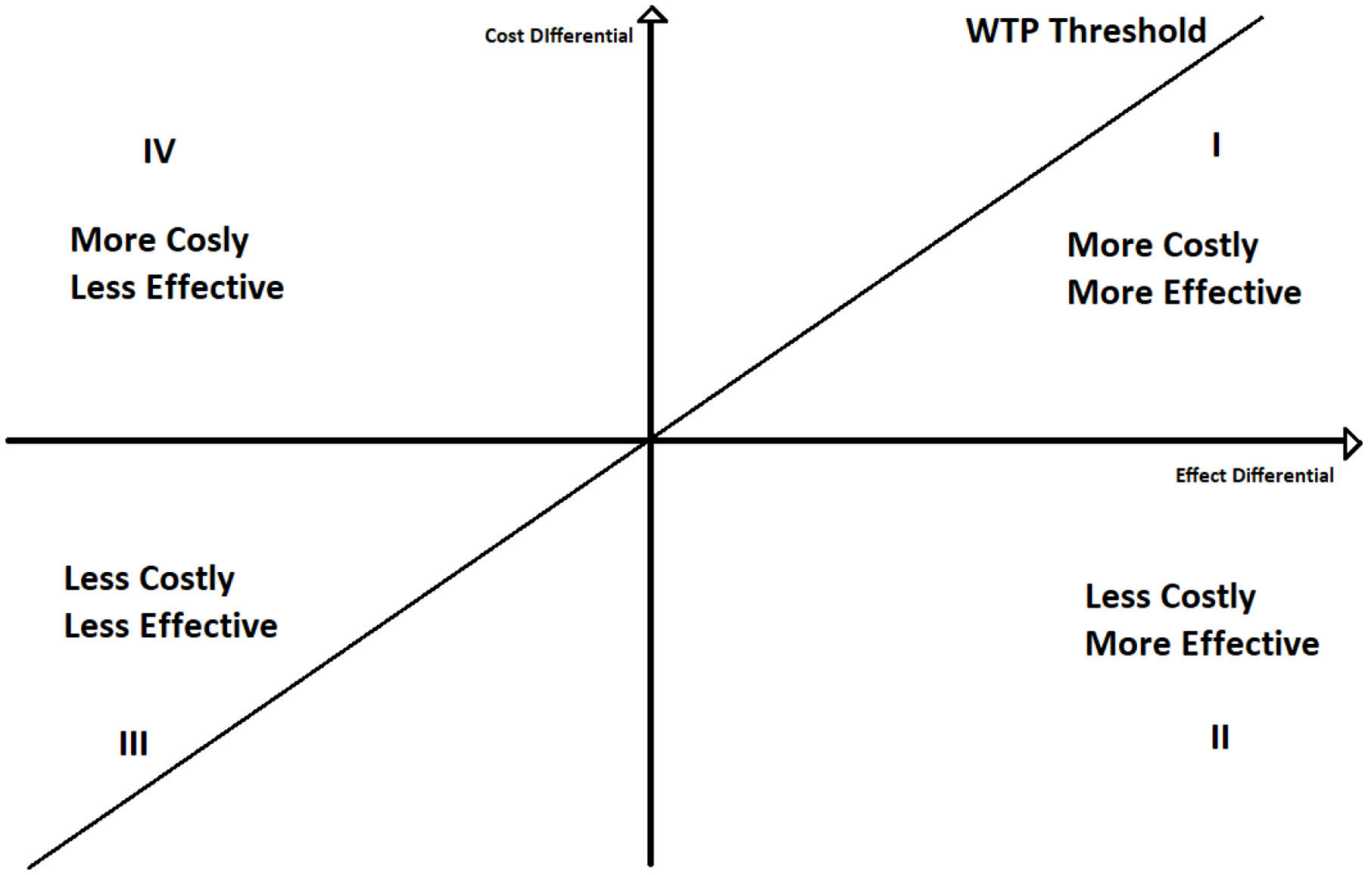
Incremental cost effectiveness ratio: four quadrant representation.

To better understand the ICER decision rule, we can plot the ICER in a two-dimensional space (Figure 1), where on the y-axis we represent the differences between the cost of the two policies, and on the x-axis the differences between their effects.

If ICER lays under the willingness to pay threshold line, policy *A* is cost effective respect to policy *B*. Moreover, if it lays on the second quadrant *A* still dominates B. Contrariwise, if ICER lays above the WTP line *A* is not cost effective, whilst if ICER is in the fourth quadrant *A* is dominated by *B*. The use of the willingness to pay thresholds is further discussed in section 4.

The objective of this paper is to show the characteristics of pharmacoeconomic studies and models, with a particular focus on Markov and Semi-Markov models. Using a cost utility model for a vaccination program for Dengue fever, we show how these kinds of models work and what are their limits. We also raise the attention on incorrect hypothesis specification and incorrect interpretation of the outcomes. Moreover, we suggest the use of some tools to bypass the limits of this approach and give some recommendations for further research. The remainder of the paper is structured as follows. Section 2 explains the theoretical background of pharmacoeconomic models and their critiques in literature. Section 3 exploits a Semi Markov model cost utility analysis for a vaccination program, which serves as background for a more accurate discussion in section 4. Section 5 ends the paper with some concluding remarks.

## 2. Methodology and Main Pharmacoeconomic Studies

In literature, there are three main types of statistical approaches to the implementation of pharmacoeconomic studies (4): regression models, decision trees, and Markov chain models. This paper will primarily focus on the latter.

The most important advantage of regression models is their capability to use least-squares linear regression techniques to explore the marginal impact of covariates on incremental cost-effectiveness instead of the usual models that aggregate cost and effect differences (5). Linear regression has been primarily used by various researchers to compute the net monetary benefit (NMB) and the net health benefit (NHM) (6–9) but it has also been used as an epidemiological model by interpreting the estimated coefficients as the risk factor weights (4). Using these models, it is straightforward to increase the number of explanatory variables in order to examine their influence on cost-effectiveness directly.

**Figure 2:**
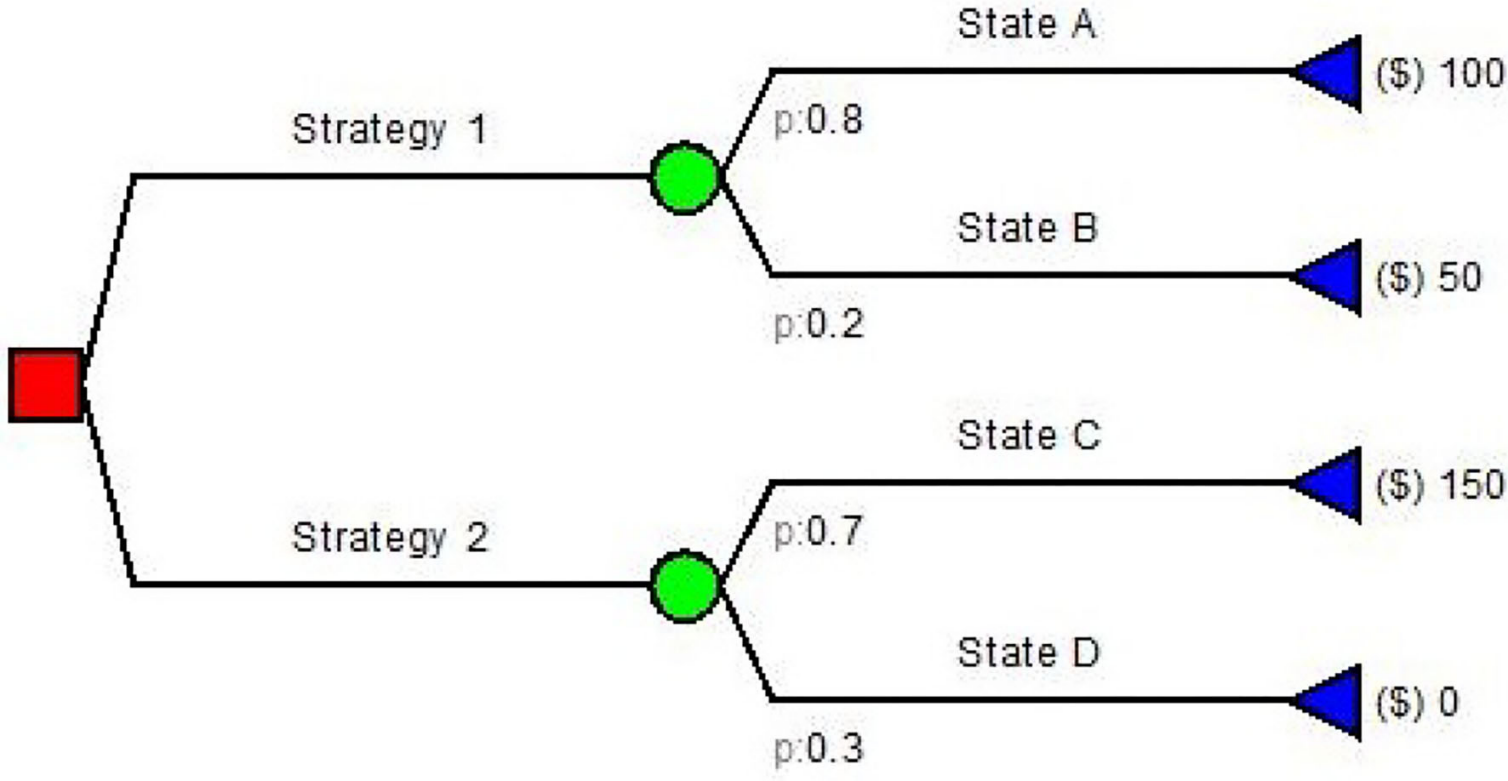
An example of Decision Tree diagram.

Decision trees are among the simplest model used in pharmacoeconomics. They are simple directed graphs without recursion (Figure 2) and represent a sequence of chance events and decisions overtime (10, 11).

Decision trees have been used for many health care problems: Caekelbergh et al. (12) tested the cost-effectiveness of a methyl aminolaevulinate-based photodynamic therapy in actinic keratosis and basal cell carcinoma, while Bachmann (13) estimated the cost effectiveness of community-based therapeutic care for children with severe acute malnutrition. Simple decision trees usually follow the same paradigm: (a) the decision node; (b) the decision strategy; (c) the outcome nodes. There is no decision if no value is assigned to the outcomes. Both input probabilities and values in decision trees are generally obtained from literature, guidelines and experts (4). If the conditions and/or data inputs evolve in time, Markov modeling is often employed as it has been designed to model these types of changes in time.

**Figure 3:**
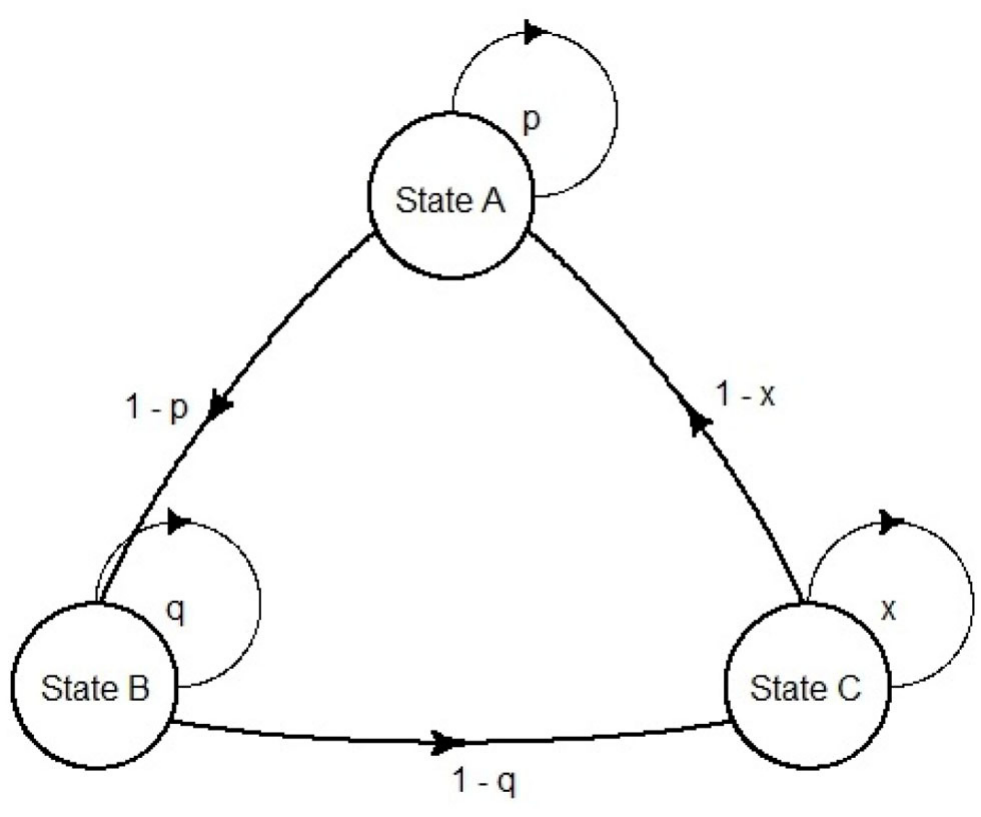
An example of Markov model transition diagram.

Markov models were first developed by the Russian scientist Andrei Markov (1856–1922). They are represented as partially cyclic directed graphs (Figure 3).

In the field of pharmacoeconomic analysis, they are exceptionally suited for diseases that involve an ongoing over time risk (e.g., risk of hemorrhage, risk of kidney failure or risk of mortality). The ongoing risk leads to important consequences: first, the time when events occur is unknown; second, an event can happen more than once, thus it is difficult in this case to use a decision tree (11). In pharmacoeconomic Markov models, health statuses are represented as Markov states, and the health changes as transition probabilities between states. Although continuous time Markov models can be built [e.g., Castelli et al. (14) use a continuous time semi-Markov model to compute cost effectiveness of two follow-up strategies in a colorectal cancer study], usually the use of discrete time Markov models is predominant in health economics (15). Although modeling continuous time is somehow required, discrete event simulation (DES) models are often suggested. DES is a flexible modeling method where entities may interact with each other for resources in a system, and each interaction between entities is an event (15). A DES approach, in contrast to Markov modeling, offers some advantages: retention of patient history; risk profile update after each event and time flexibility. In view of that, although in Markov models the length of a cycle is fixed, in DES the simulation time can adapt directly to the time the next event occurs (16). Markov models compensate their limitations with their simplicity, as they can be visually inspected for programming errors, and can be tested straightforwardly for technical replication (17).

Notationally, a Markov chain is a sequence of random variables *X*_1_, *X*_2_, ..., *X*_*n*_, ... characterized by the Markov property (Equation 2), which states that a model is memoryless: the conditional probability of the forthcoming state depends on the current state only, thus it does not depend on the previous ones (18, 19). This means that, for all *n* and states *x*_*n*_

(2) $$\begin{array}{l} \begin{array}{c} \mathrm{Prob}\left\{X_{n+1}=x_{n+1}|X_{n}=x_{n},X_{n-1}=x_{n-1},\ldots ,X_{0}=x_{0}\right\}\\ \text{ Pr}\left\{X_{n+1}=x_{n+1}|X_{n}=x_{n}\right\} \end{array} \end{array}$$

Let us define *S* = *s*_1_, *s*_2_, ..., *s*_*r*_ of *X*_*n*_ as the set of possible states. This set is defined as the state space of the chain. The chain moves between states and the probability *p*_*ij*_ to move from state *s*_*i*_ to state *s*_*j*_ is called the “transition probability.” It expresses the conditional probability of making a transition from the state *x*_*n*_ = *s*_*i*_ to the state *x*_*n*+1_ = *s*_*j*_, when time moves from *n* to *n* + 1. Transition probabilities are defined as

(3) $$\begin{array}{l} p_{ij}\left(n\right)=\mathrm{Pr}\left\{X_{n+1}=s_{j}|X_{n}=s_{i}\right\} \end{array}$$

The matrix *P*(*n*) = (*p*_*ij*_)_*ij*_, created by quantifying *p*_*ij*_ in row *i* and column *j*, for all *i* and *j*, is named the “transition probability matrix” or “chain matrix.” All the elements of *P*(*n*) satisfy the following properties:

(4) $$\begin{array}{l} 0\leq p_{ij}\leq 1 \end{array}$$

and, for all i,

(5) $$\begin{array}{l} \underset{j}{\sum }p_{ij}=1 \end{array}$$

Usually, Markov models are time-homogenous. This means that there are no changes in the transition probabilities as time goes on, but in modeling health care generally non-homogenous Markov models (also called Semi-Markov models) are used (15). In this case, the transition probabilities depend upon the amount of time that has passed. In both cases (homogeneous or non-homogeneous models) the process may, or may not, be stationary. A process is said to be stationary if it is invariant under an arbitrary shift of the time origin (18). A discrete time Markov model consists of one or more communicating classes that form a set of states that communicate. If the chain is composed of one communicating class only, the chain is said to be irreducible (19). If the transition probability is *p*_*ii*_ = 1, the state *s*_*i*_ is defined as an absorption state, which corresponds to a closed communicating class. A typical example of absorption state in health care Markov model is “death.”

In order to use a Markov model in a pharmacoeconomic study, it is fundamental to attach weights to the states, that allow the analyst to estimate cost and health outcomes. As an example, for predicting life expectancy a zero weight is attached to the death state and a unit weight is attached to the other states. Running the model for many cycles provides an estimate of the average life expectancy. In the case of an economic evaluation, researchers are interested in the quality adjusted life year, or in the effect of a therapy. Thus, these kinds of elements need to be attached in a similar way to the life-expectancy case. On the cost side, the model behaves likely: the costs of spending one cycle in each of the states are assigned to that state and, as the model runs for many cycles, the total cost is obtained by summing across those cycles (20). Moreover, to make this economic model more realistic, adjustments for differential time are needed. These adjustments are done by discounting outcomes and costs. This allows the user to compare costs and outcomes in terms of a net present value. In the pharmacoeconomic literature, the use of Markov models or Semi Markov models is vast and growing. Anis et al. (21) constructed a Markov model with the objective to attain a CEA for antiretroviral therapy on HIV-positive patients. Leelahavarong et al. (22), on the other hand, tried to identify through a Semi-Markov model the maximum price at which HIV vaccination is cost-effective, comparing it with the normal HIV prevention programs in a governmental prospective. Although Markov models can be used for infectious disease modeling, dynamic models are better suited for the task. For instance, DePasse et al. (23) modeled a CEA with an agent based approach for an influenza vaccination campaign in the U.S. Moreover, the two approaches can be used simultaneously. As an example, in Khazeni et al. (24), in which the cost-utility of pandemic influenza vaccination intervention is quantified in terms of QALY through a joint use of a compartmental epidemic model and a Markov model of disease progression. Furthermore, Yaesoubi and Cohen (25) and Haeussler et al. (26) use a Markov model with a force of infection function that accounts for time dependent changes in prevalence and, consequently, for the effects of herd immunity. With this approach, in contrast to the deterministic compartmental models, it is possible to approximate the spread of the disease in large populations with a small state-space size, controlling for both an acceptable degree of accuracy and computational time. Although Markov models sometimes can be applied for infectious disease modeling, they are also suitable for modeling pharmacoeconomics for transplant, as in Jensen et al. (27) and in Rodina et al. (28). Both studies conducted a CUA for kidney transplant compared to dialysis.

An intensive use of Markov models can also be observed in pharmacogenomics testing and precision medicine: by doing a genetic test to a patient, it is possible to formulate a personalized and more successful therapy. Therefore, in theory the cost of the test should be counterbalanced by the higher effects of the therapy. Examples of this kind of models are genetic testing for major depressive disorder (29), cardiovascular prevention (30), and epilepsy (31).

Moreover, Markov Models for economic evaluation of healthcare interventions are particularly suitable for modeling the cost effectiveness of new health care interventions for non-communicable parasitic diseases. Examples of these diseases are Dengue fever, West Nile fever, Changa disease, and Malaria. These diseases are such that the parasite life cycle does not include direct host-to-host transmission, thus they are not contagious directly among humans (32). Studies on intervention for these diseases are those of Shankar et al. (33) and Lee et al. (34) with respect to a vaccination program for West Nile fever. Finally, Seo et al. (35) compare the cost effectiveness of a vaccine for Malaria in comparison with long lasting insecticide treated nets. In the framework of Dengue fever, many papers using Markov models have been proposed in the literature for economic evaluation of healthcare interventions. Valuable studies are that of Perera et al. (36), which analyzes from a societal perspective the cost utility of the CYD-TDV vaccine and a pre-vaccination serological screening for Dengue fever in Sri Lanka; Orellano et al. (37) estimate the cost-utility of a Dengue vaccine in a country with heterogeneous risk of Dengue transmission using the incremental cost effectiveness ratio for DALY averted as outcome; Lee at el. (38), on the other hand, analyze the economic value of Dengue vaccine in Thailand for various level of vaccine efficacy; Shepard et al. (39, 40) carry out similar analysis but for a hypothetical pediatric Dengue vaccine in South Est Asia and Panama; Fitzpatrick et al. (41) run two probabilistic Markov chains in parallel for vector and human populations, where the probability of a vector being infected with the Dengue virus depends on the number of infected humans and the probability of a human to be infected depends on the number of infected vectors.Beside Markov models, which in their standard version are static models and cannot capture indirect effects such herd immunity or increase transmissibility, recent economic evaluations of interventions on Dengue fever often use dynamic transmission models, described for instance in Flasche et al. (42): four deterministic compartmental models and four stochastic simulation models are compared among each other to evaluate the cost-effectiveness of a Dengue vaccine (CYD-TDV; Dengvaxia) in Latin America and Southeast Asia (43, 44). Moreover, Lee et al. (45) compare the cost-effectiveness of the CYT-TDV vaccine respect to an hypothetical new Dengue vaccine using a spatially explicit individual-based transmission model.

## 3. Simulation Experiment

In this section, we show some examples to demonstrate how and why Markov models are often used in pharmacoeconomic analysis. Our analyses are carried out using the R software for statistical computing (46), and in particular, the package Heemod (47).

Since our main aim is to show how Markov models work in Pharmacoeconomics, we exploit a semi-Markov model on the cost utility of a vaccine for the Dengue fever disease. This is a simplified version of the model described in (36) which analyzes, from a societal perspective, a Dengue (CYD-TDV) vaccination program following a pre-vaccination serological screening in Sri Lanka. Since the main purpose of our analyses is showing pros and cons of semi-Markov models in Pharmacoeconomics, we do not consider the part of the model involving screening. Our model, moreover, does not take into account the herd effect resulted from the dengue vaccination campaign nor the vaccine efficacy waning, as well as it does not deal with the well-known CYT-TDV safety issues (48), as we do not consider the serological screening as in Perera et al. (36). However, these limitations do not undermine the relevance of our study. The semi-Markov model we use for Dengue is just an instrument to emphasize advantages and disadvantages of Markov models in Pharmacoeconomics and to highlight the critical points that need to be overlooked to pursue rational health care policy decisions.

### 3.1. Model Inputs

Cost, utilities, and transition probabilities of Dengue fever disease, retrieved from literature, are reported in Table 1 with the related references in the last column.

**Table 1:** Parameters of the model.

**Data input parameter**	**Value (range)**	**Distribution**	**References**
Dengue incidence	0.0088 (0.007–0.0106)^a^	Binomial	(36)
Age-specific risk of DF	1 − exp(−0.000259·age^3.991^)		(37)
Probability to have DHF if DF	0.4342 (0.347–0.521)^a^	Binomial	(36)
Probability of Death if DHF	0.0053 (0.0042–0.0064)^a^	Binomial	(36)
Vaccine efficacy against DF	0.647 (0.587–0.0698)^b^	*Beta*(143, 78)	(37)
Vaccine efficacy against DHF	0.955 (0.688–0.999)^b^	*Beta*(5, 0.24)	(37)
Cost of DF	$51.4 (41.08–61.62)^a^	Gamma, *sd* ± 3	(36)
Cost of DHF	$143.46 (112–169.2)^a^	Gamma, *sd* ± 6	(36)
Cost of Death	$3000 (2400–3600)^a^	Gamma, *sd* ± 10	(49)
Cost of Vaccine	$73 (58.4–87.6)^a^	Gamma, *sd* ± 3	(36)
Disability weight for DF	0.197 (0.172–0.211)^c^	*Beta*(19.7, 80.3)	(36)
Disability weight for DHF	0.545 (0.475–0.583)^c^	*Beta*(54.5, 45.5)	(36)
Discount rate of cost	0.03	Point estimate	(36)
Discount rate of benefit	0.015	Point estimate	(50)

a * Range: value ±20%*.

b *Range: 95% CI*.

c *Range: minimum-maximum*.

**Figure 4:**
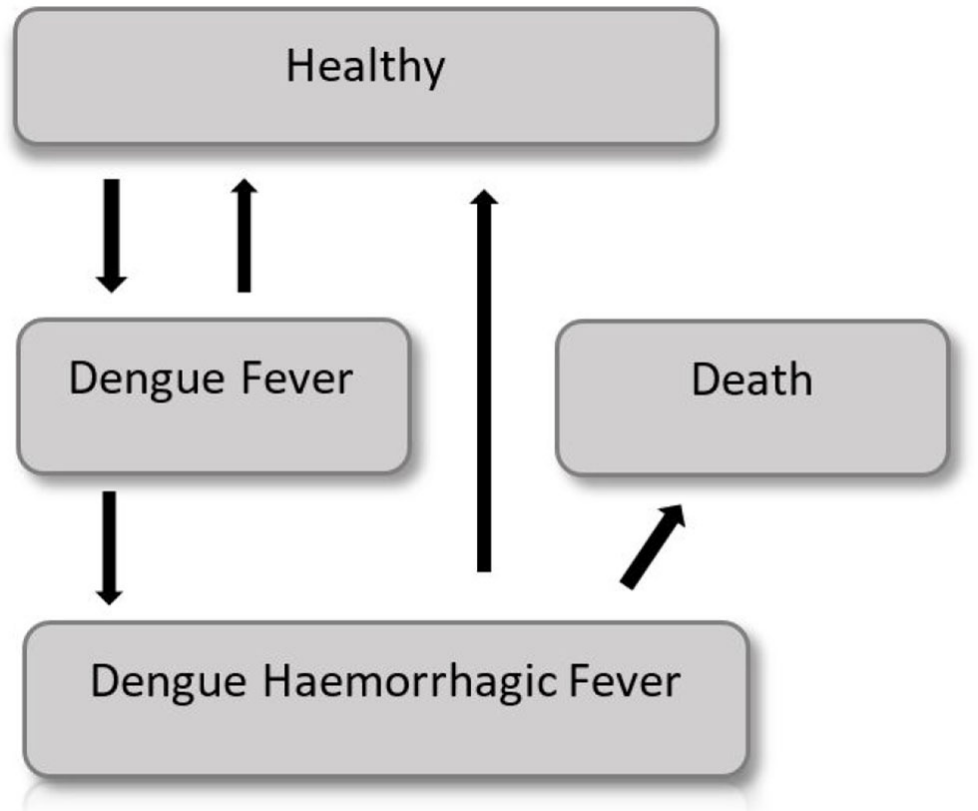
The semi Markov model for the Dengue fever disease.

The probability of showing the Dengue symptoms is correlated with the age of the cohort and thus a semi-Markov model is considered. A graphical representation of the model is shown in Figure 4.

Costs and utilities, in form of QALYs, are attached to each of the Markov states, and a discount rate is applied for both benefits and costs. A time horizon of 10 years is used for the cost utility analysis. Two identical cohorts of 1,000 9-years-old children compose both the intervention and control groups.

### 3.2. Base Case

Early in the disease the entire population is in a healthy state, but each year (corresponding to a Markov cycle) there is a chance to get sick and to show Dengue fever symptoms, which require moderate medical attention. Subjects with Dengue fever can either get cured, and go back in the healthy state, or worse their condition and show a Dengue hemorrhagic fever (DHF), which requires intensive care. DHF patients have a certain probability to die, or they can recover and go back to the healthy state. The probability to show symptoms changes according to the age of the cohort. Thus, it changes with time making this model a non-homogeneous Markov model. The difference between the intervention group and the control group is the use of a vaccine, which lowers by a coefficient (efficiency of the vaccine) the probability to develop the Dengue fever or the DHF.

### 3.3. Sensitivity Analysis

**Figure 5:**
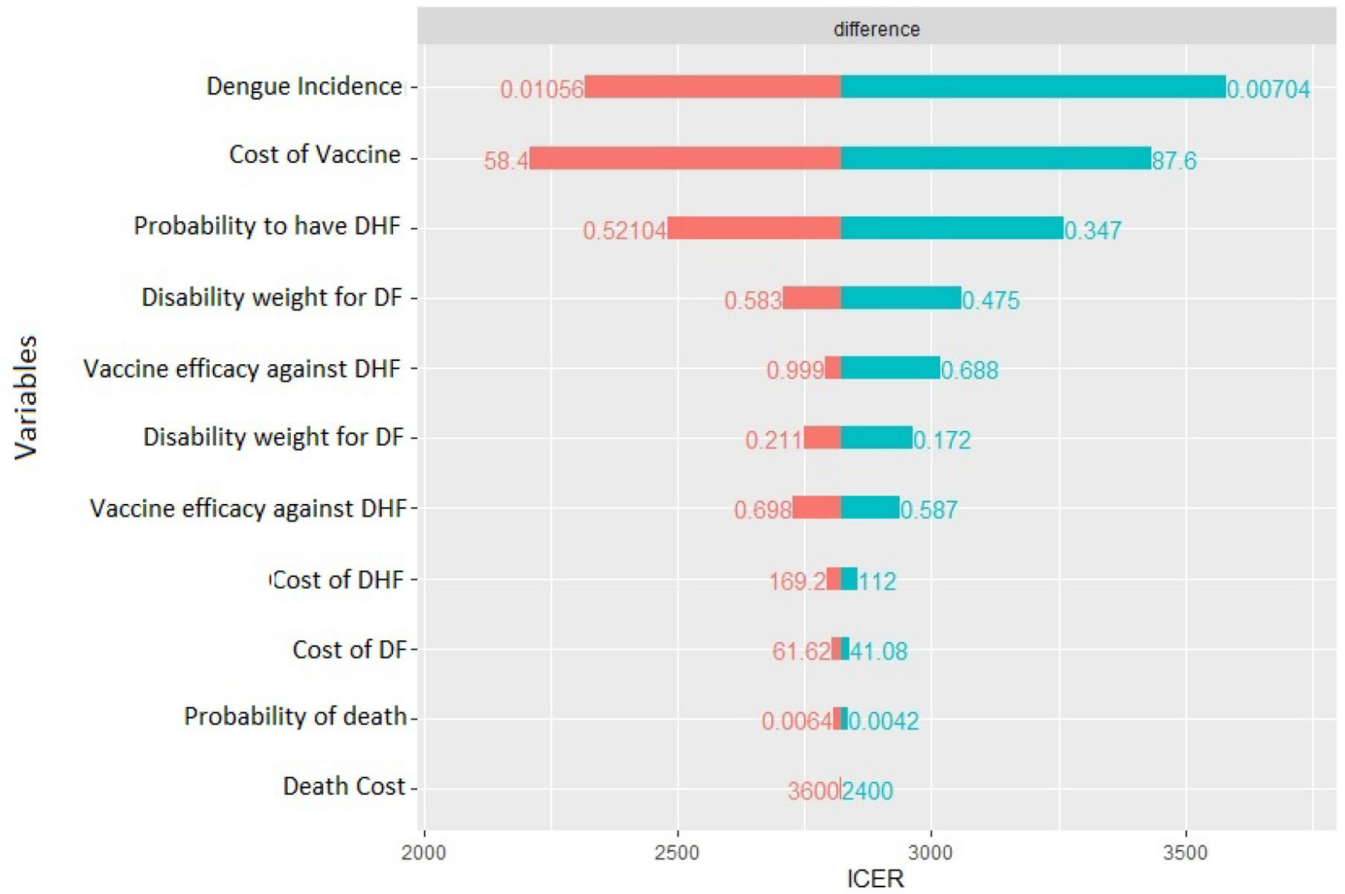
Tornado diagram of deterministic sensitivity analysis.

We perform a deterministic sensitivity analysis to check the uncertainty of each parameter. Whenever possible, we use upper and lower bound of the 95% percentile of the distribution of a specific parameter, otherwise we use the ±20% value, or the minimum-maximum range found in literature (37). Therefore, each time one parameter is varied by a higher and a lower value with respect to the base case within a certain range. The result of this analysis is the range of the ICER due to parameter uncertainty. The tornado diagram (see Figure 5, next section) summarizes the results. In order to assess how changes of several variables affect the ICER, a probabilistic sensitivity analysis (PSA) based on 1,000 Monte Carlo simulations is also performed. This is done by choosing a random value from the distribution of the parameters that are uncertain. Probabilities of Dengue Incidence, of having DHF as well as of dying because of DHF follow a binomial distribution, utilities and vaccine efficiency follow a beta distribution, while costs follow gamma distribution (see Table 1) (51).

**Figure 6:**
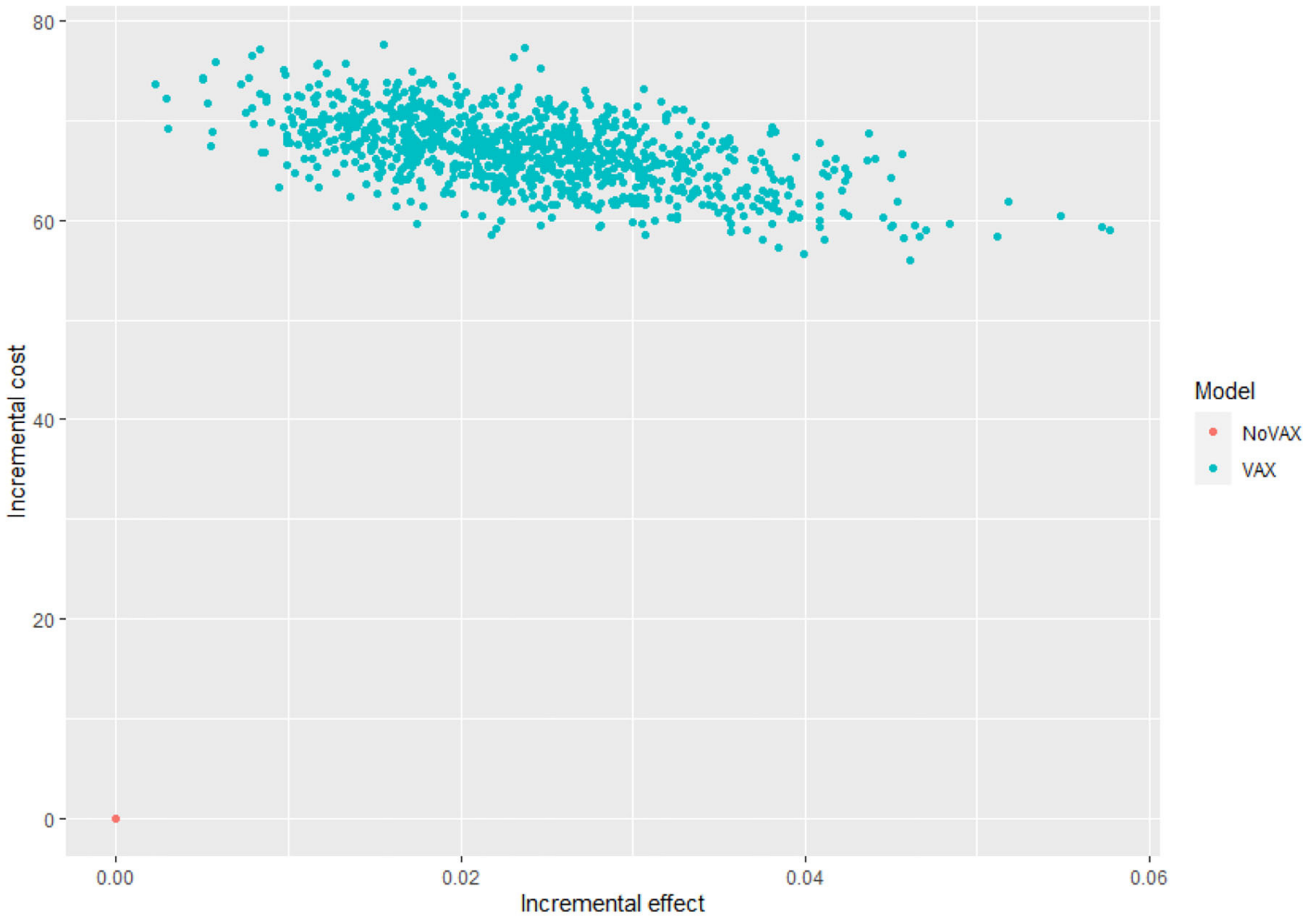
Cost effectiveness plane.

**Figure 7:**
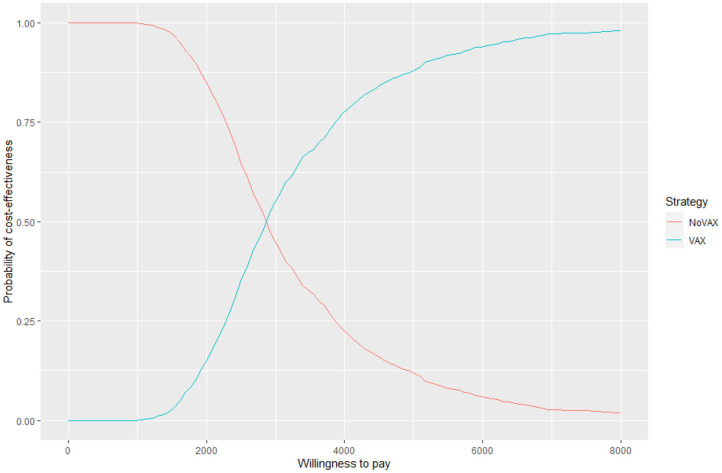
Cost effectiveness acceptability curve.

For each iteration, incremental costs and incremental QALYs are computed. The results of these iterations are shown in a cost-effectiveness plane (CE-plane) (see Figure 6, next section). Based on the CE-plane, it is also possible to define a cost-effectiveness acceptability (CEA) curve (see Figure 7, next section). The CEA curve shows the probability that vaccination is cost-effective compared to the no vaccination control group for different threshold values of willingness-to-pay per QALY gained.

### 3.4. Results

The base case analysis, whose results are presented in Table 2, shows that the vaccination results in an incremental cost of 67.01US$ per patient, and an incremental QALY of 0.0238 US$ per patient, causing an ICER of 2820.95US$ per QALY gained.

**Table 2:** Base case results.

**Strategy**	**Costs**	**Δ costs**	**QALY**	**Δ QALY**	**ICER**
Vax	$74.26	$67.01	9.355	0.0238	$2820.95
No Vax	$7.24		9.331		

The deterministic sensitivity analysis shows the relative contribution of each parameter for the estimation of ICER. The diagram in Figure 5 shows which of the parameter can have the greatest contribution.

In our case, the risk of getting the Dengue fever is the one that has the major impact, a higher risk lowers ICER, but still it cannot result in a cost-dominant scenario (less costs, more QALY). The same can be said for the probability to worse from the Dengue fever to DHF. Moreover, the cost of vaccine is an important cost driver, which can remarkably lower the incremental cost utility ratio. The probability to die for Dengue, the other costs, and the disability weights compared with the other parameters do not affect highly the decision of this specific health care policy: higher or lower values of these parameters do not lead to high differences in results. Since the degree to which ICER is more favorable depends majorly on the incidence of the Dengue, in countries or regions where the risk to get infected is higher a vaccination program is obviously recommended.

The Monte Carlo simulation assesses the uncertainty surrounding the point estimates of the base case scenario. The results are show in Table 3 together with their 95% confidence interval.

**Table 3:** Results of the probabilistic sensitivity analysis.

**Strategy**	**Costs (95% CI)**	**Δ costs (95% CI)**	**QALY (95% CI)**	**Δ QALY (95% CI)**	**ICER**
Vax	$74.16 (69.42–79.04)	$66.9 (60.89–72.65)	9.355 (9.351–9.358)	0.0238 (0.0112–0.0383)	$2812.72
No Vax	$7.26 (3.37–11.39)		9.332 (9.314–9.347)		

The results show that ICER is 2812.72US$. This value is very similar to that of the base case scenario, as the difference per patient is $66.9 in terms of cost, and 0.0238 in terms of QALY.

Even if these results are meaningful, the most useful way to express uncertainty about cost utility is through the cost effectiveness acceptability curve and the cost effectiveness plane (52) (Figures 6, 7).

Figure 6 shows the cost effectiveness plane, where each dot represents a Monte Carlo iteration of PSA. It seems that incremental costs are rather stable, whist the incremental effect is more uncertain. With this information we can derive Figure 7, which shows the probability to have an ICER greater than a certain threshold, i.e., the willingness to pay (WTP) per QALY gained. The higher is the threshold, the higher is the probability that the vaccine is cost-effective. If the WTP threshold is 3,000 US$, more than half of the simulations show an ICER lower than this value.

**Figure 8:**
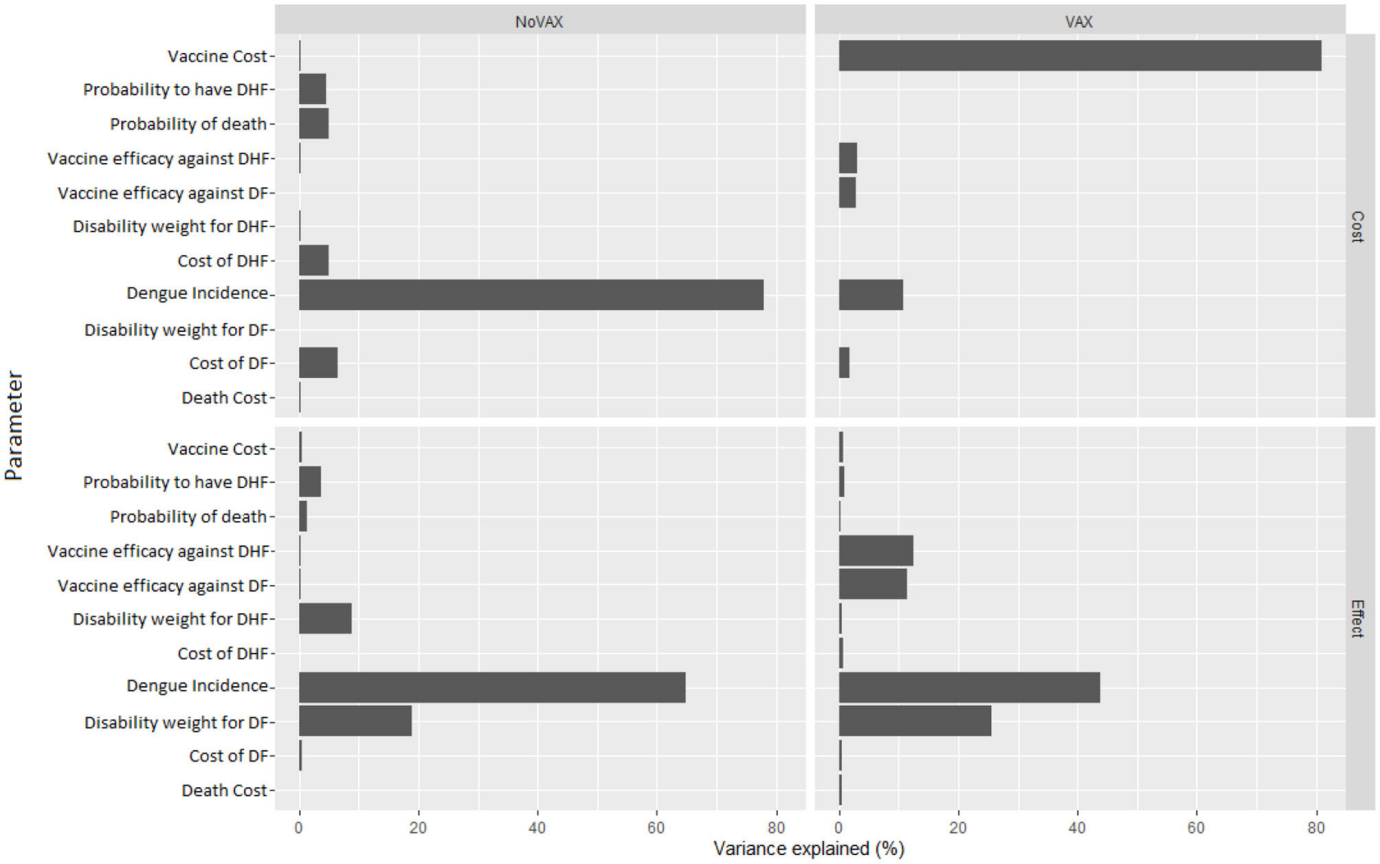
Covariance analysis of PSA result.

Figure 8, instead, represents the covariance analysis of PSA. For each strategy, it shows the proportion of PSA variation explained by each parameter, for both costs and effects. In our model, the key parameters are the vaccine cost and the incidence of Dengue fever, whilst the cost of death does not influence PSA relevantly.

Finally, we assess if the policy causes important variation of ICER as well as we determine the most important parameter that have to be kept in higher consideration. Results about variations of estimated parameters and subsequent ICER values are reported in Table 4, which is self-explanatory.

**Table 4:** Parameters and ICER range.

**Parameter**	**Value range**	**ICER range**
Death cost	(2400–3600)	(2823.735–2818.15)
Cost of Dengue fever	(41.08–61.62)	(2839.24–2802.855)
Cost of DHF	(112–169.2)	(3580.01–3025.52)
Cost of vaccine	(58.4–87.6)	(2206.358–3435.528)
Disability weight for DF	(0.172–0.211)	(2962.040–2747.64)
Disability weight for DHF	(0.475–0.583)	(3058.665–2706.74)
Vaccine efficacy against DF	(0.587–0.698)	(2938.149–2727.824)
Vaccine efficacy against DHF	(0.688–0.999)	(3018.16–2790.69)
Dengue incidence	(0.00704–0.01056)	(3580.014–2314.89)
Probability to have DHF	(0.347–0.521)	(3260.042–2480.894)
Probability of death	(0.0042–0.0064)	(2835.298–2806.703)

Nevertheless, there are more factors, beside the model inputs, that require more focus. These factors, that include inputs and outputs of the model, together with tools to bypass some model limitations are individually discussed in the next section.

## 4. Discussion

The example shown in the previous section is aimed at providing an overview about how Markov models work in Pharmacoeconomics. In this section, we emphasize some critical points of these models. Particularly, we take a closer look at the incremental cost effectiveness ratio, the willingness to pay thresholds, the model life span, the size of the cohorts, as well as at two known problems of Markov models: the increasing complexity of the model arising when many states are considered and the memoryless condition.

### 4.1. Incremental Cost Effectiveness Ratio. Which Measure?

The main outcome of any cost utility model is ICER. It tells the policy makers if the health care policy is worth to be financially supported or not. In the previously reported model ICER has been computed using the mean costs and the mean QALY of the simulation. Another way to compute ICER is using the median cost and median effect. In our case, the mean ICER is slightly lower than the median ICER, the latter being 2888.36US$. The difference between the two measures is 75.66US$ only. Bang and Zao (53) suggest that mean and median based ICERs must be considered together as complementary tools in the cost effectiveness analysis for an informed decision, acknowledging the pros and cons of each.

### 4.2. WTP Thresholds. Is a Policy Really Cost-Effective?

ICER is an absolute measure, thus it must be compared to some thresholds. Indeed, the decision rule mainly depends on the willingness to pay per QALY gained thresholds. These thresholds reflect health opportunity costs and are generally used to assess whether an intervention is worth. Nevertheless, these thresholds differ among countries and among the kinds of health care programs and their corresponding perspective. Wood et al. (54) reject the guidelines of the World Health Organization (WHO) because they tend to suggest too large thresholds (up to three times the GDP per capita) and, in contrast, they estimate thresholds as 1–51% of the GDP per capita for low/middle income countries and 18–71% for middle/high income countries. In the model discussed in the previous sections, we examine the use of a vaccine in Sri Lanka. The 2018 GDP per capita of this country, according to the World Bank, is 4102.48US$. Thus, following Wood et al. (54), with an ICER of 2812.72US$ we are not able to give a clear answer about the possibility of funding this policy. Rather, if we follow WHO which tends to suggest larger thresholds, we can say that the policy is worth to be funded.

### 4.3. How Does the Cost of the Vaccine Influence the ICER?

**Figure 9:**
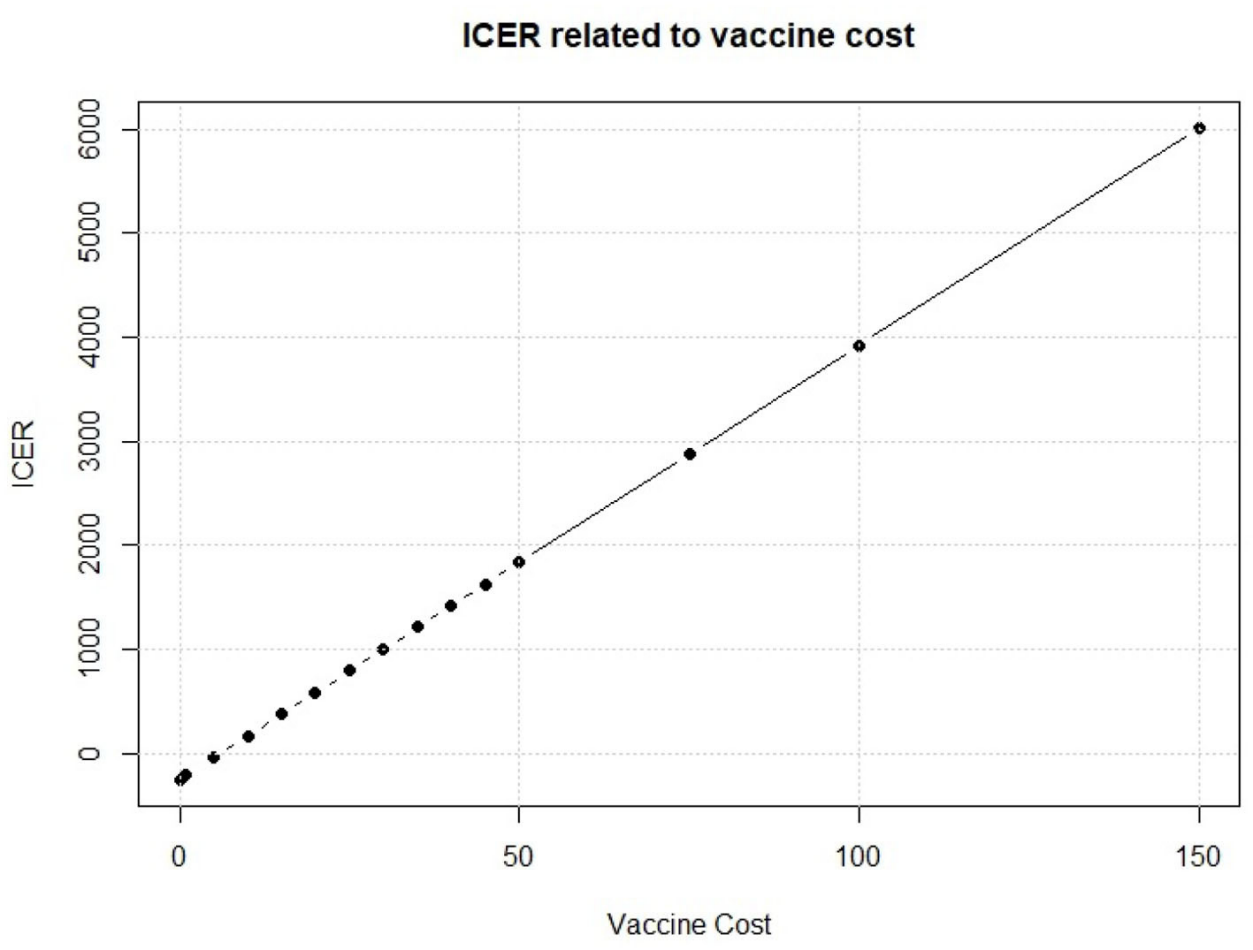
Relationship between vaccine cost and ICER.

We see in Figure 8 that, on the cost side, the greatest part of variability of the vaccination program in the PSA is explained by the cost of the vaccine. To further investigate about this issue, we fix the vaccine cost in each interaction of the Monte Carlo simulation of the PSA. We run the simulation for several possible values of the vaccine cost. Results of this fixed-cost vaccination Monte Carlo Simulation are shown in Figure 9.

An increase in the vaccine cost leads to a linear increase of ICER. The break-even point, i.e., the point when the vaccine strategy changes from cost efficient to dominant, is observed for a vaccine cost of 5.998$ which leads to an ICER of 0.01$ for QALY gained. This means that the vaccine intervention will not increase the total cost respect to the standard no intervention program, but it will provide an increase in QALYs for the population of interest. Thus, it will be rationally founded whichever the optimal threshold is. Moreover, a vaccine that would cost < $6 provides a negative ICER, making the vaccination program dominant, with a decrease of total cost but an increase in effect. Policy makers, thus, should identify the key parameters that they can somehow control, like the vaccine cost, and try to drive their value in favor of the new policy.

### 4.4. Model Time Span: Which Magnitude?

**Figure 10:**
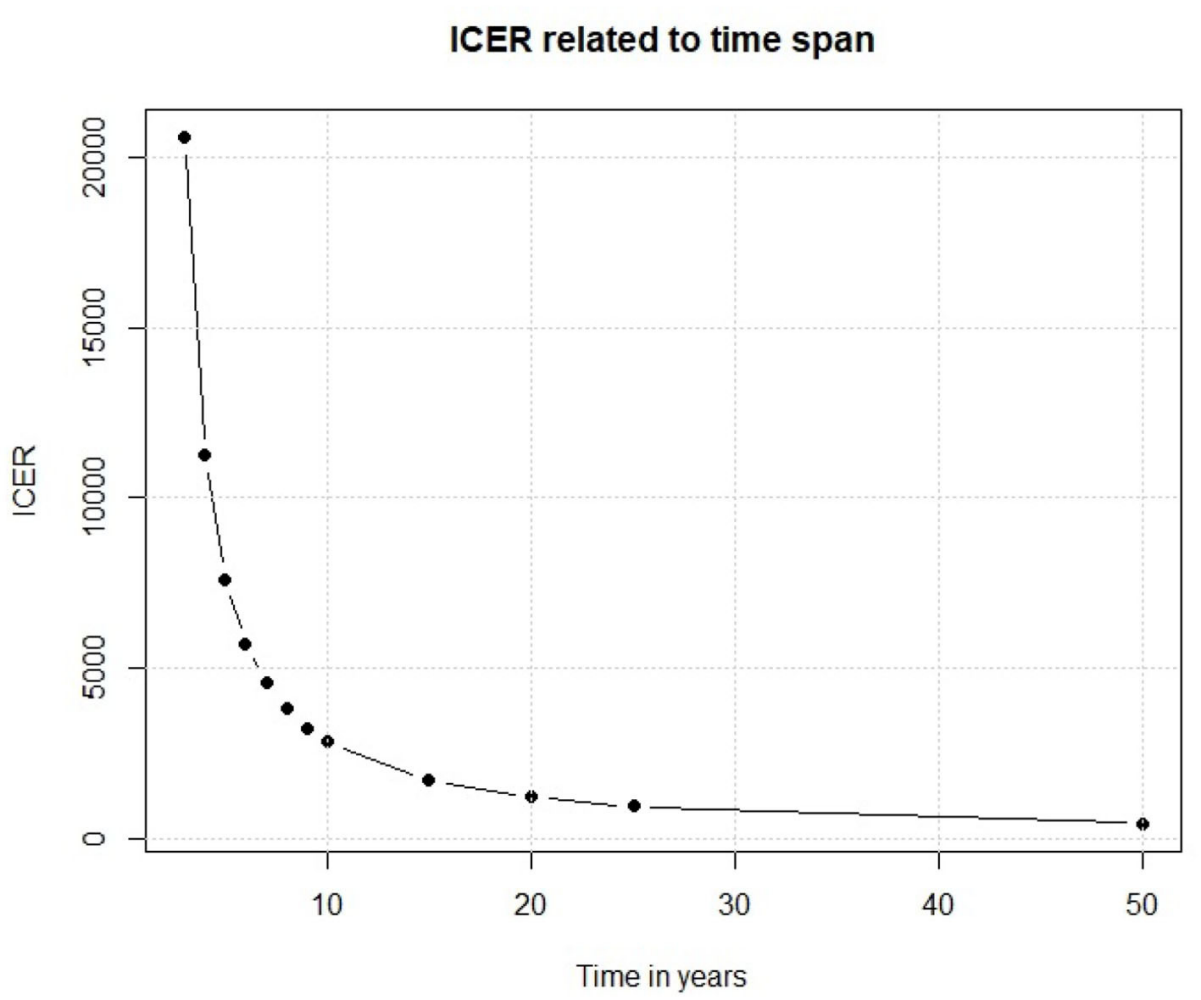
Relationship between time span cost and ICER.

We have so far examined the outputs of our Markov model. Moving to the input parameters, one of the main factor is the time interval in which the policy is analyzed. A slight change in its length could cause important changes in the outcomes. In our model, we assume that the efficacy of the vaccine would last for 10 years because of the changes over time of the serological patterns of Dengue. If this assumption is not true, and the efficacy of the vaccine lasts for much less time, the results are very different. To assess this point, we have replicated the base case scenario each time using a different time span, and from Figure 10 we observe that the relationship between time and ICER is not linear, despite the previously shown ICER-Vaccine vs. cost linear relationship.

Results reported in Figure 10 show that if the vaccine efficacy lasts only few years the policy is not enough cost efficient for a middle/low income country like Sri Lanka. On the other side, if the vaccine efficacy lasts longer we observe an important decrease in ICER, which eases the policy decisions. This simple experiment shows that, before discussing a policy of this kind, there are important factors that need to be considered. Thus, the scientific foundations of the study should be very solid.

### 4.5. Size of the Cohorts: How Many Participants Are Needed?

In literature [see e.g., (55)] it is reported that in observational studies it is essential to calculate the required sample size in the design phase. If the sample is too small it may produce an estimate for the population prevalence of an infection that is too imprecise to be useful for public health planning and prevention. Too large a sample may have used too many resources and taken an unacceptably long time to collect. In our simulations, we consider two identical cohorts of 1,000 9-years-old children for both the intervention and control group. Indeed, unreported outcomes provides evidence that applying the same Markov model with cohorts of children of different sizes, ranging from very small sample sizes of 100 units to extremely large ones (10,000 units) does not change significantly the results provided by the model. These remain practically unchanged. This indifference to the sample size seems to be another limitation of these class of models.

### 4.6. Number of States: How Much Complex the Model Need to Be?

Markov Chain models, although they often work fine, have some limitations about their use. One of the main problems is that they become very complicated when more states and more interactions among states are included. This complexity becomes particularly problematic in presence of time-dependent probabilities. One can take the model discussed in the previous sections as a reference. In the case a screening with a Dengue immunoglobulin (IgG) antibody test to detect the serostatus of the population of interest needs to be added to the vaccine policy, many more Markov states have to be included into the original model to take into account IgG positives and negatives, as well as the sensitivity and specificity of the test itself. In this scenario, it would be also useful to differentiate between sick patient hospitalized and in intensive care, considering the different costs and utilities. This specification would require adding even more Markov states to keep these differences into consideration. Adding more and more conditions causes the transition matrix to be very large, leading to a more complicated model, which is quite hard to manage.

### 4.7. Tunnel States vs. Time Varying Probabilities: Which Model to Choose?

**Figure 11:**
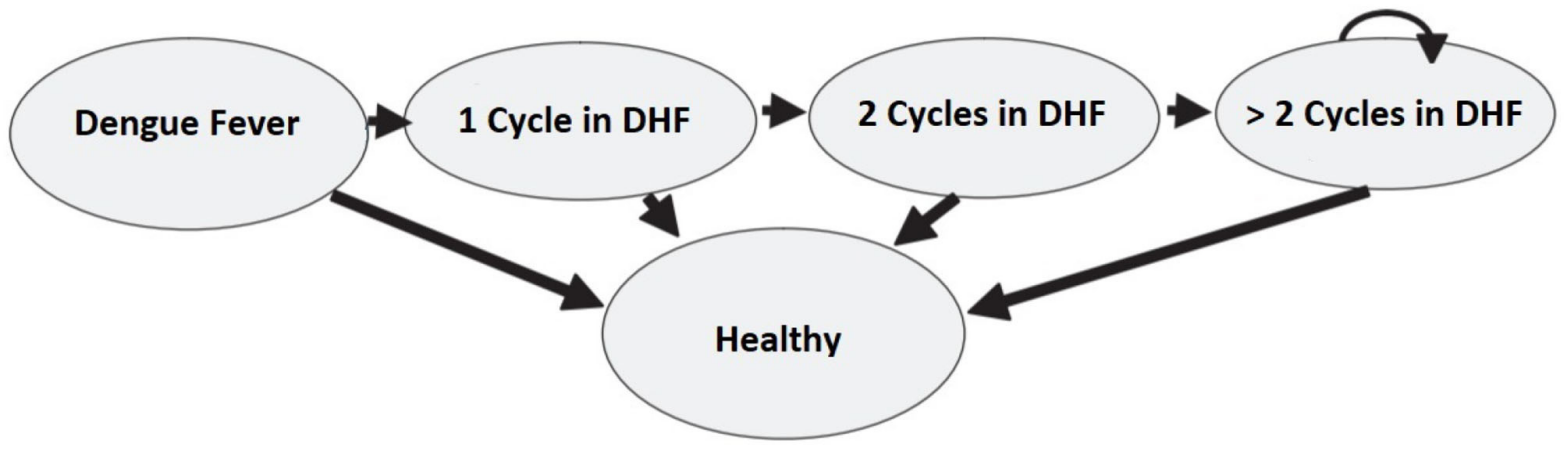
Example of the use of tunnel states in a Dengue fever model.

Another limitation of the Markov Chain models is the lack of memory. In fact, the probability of moving between states does not depend on the previous cycles (56). To overcome this limitation, Hawkins et al. (57) suggest using “tunnel states” that enable to integrate the health experience from the previous Markov cycles. The term “tunnel” implies that the states of the cycles can be assessed only in a pre-determined sequence, as passing through a tunnel. This method can be applied in the dengue fever model. Let us suppose that patients in the DHF state can stay sick for more than one cycle. This assumption is in contrast with the model presented in section 3 where patients in the DHF state could either get cured or die. Moreover, the probability to stay in the same sick state (i.e., to keep on showing DHF symptoms) is not constant as it depends on how long the patient has been sick in the past. Specifically, the probability of an improvement increases in the first two cycles, but then it remains stable in time. Figure 11 describes the model and the use of the tunnel states.

We could have obtained the same results with time varying transition probabilities. The choice between the two methods depends mainly on the type of model the researchers want to specify. There are cases when tunnel states work better, and other when a time varying probability is more appropriate. The latter can be implemented easily. In the model presented in section 3 we used a time varying probability approach in the incidence of Dengue fever in the cohort. Indeed, the incidence is correlated with the age of the population of interest, thus it changes as the time pass. Instead, the tunnel state approach would have required the use of a large number of tunnel states, yielding to a more complicated and difficult to handle model.

## 5. Concluding Remarks

Markov models are popular tools used for the evaluation of the effects of specific health care policies. In this paper, we have exploited the theory behind these particular kinds of models and have explained how they can be used for choosing the right policy to fund. Through a simulated Markov model, we have focused the attention on the key components that make this approach helpful, and on the interpretation of the results through useful tools like the tornado diagram, the cost effectiveness plane, the cost effectiveness curve and the covariance analysis of PSA results. Moreover, we have described the median ICER which is computed using the median cost and the median effect, and we have compared it with the standard mean ICER and showed that the two measures should be taken in consideration together. We have also documented that the willingness to pay threshold can vary between countries and that the WHO guidelines are not unanimously accepted in literature. Next, we have alarmingly highlighted that if the scientific bases like the duration of a vaccine efficacy or the time span of the model are not solid and robust, model outcomes can differ importantly. Finally, we have also discussed about the use of tunnel states compared to time varying transition probabilities, focusing on the pros and cons of the two approaches.

All the theoretical and practical issues presented in the paper lead us to the conclusion that it is of fundamental importance to recommend policy makers and researchers to take into account all the above-mentioned pros and cons of phamacoeconomic Markov models and thus to interpret their results with extreme caution before funding any health care policy. The correct specification of input parameters, including the time span of the study and the size of the cohorts, is of fundamental importance for the implementation of a model leading to reliable and realistic decisions in public health policy setting. Our general recommendation for future research on the use of Markov models in Pharmacoeconomics is that this model should be tested in conjunction with one or more additional approach to check if the outcomes are consistent and robust enough. Moreover, specification of input parameters and other initial information should not be taken parsimoniously from previous literature, as they could cause estimation errors and excessive approximation. It would be preferable, to obtain robust outcomes and thus take reliable decisions, that input parameters should be estimated from real data available to the researcher. This is particularly important when the distribution of input parameters needs to be inferred to carry out a sensitivity analysis. In fact, as shown in the previous section, wrong assumptions can lead to sometimes extremely wrong decisions, with a waste of financial resource, and most importantly, they can negatively affect the health of the population of interest.

## Data Availability Statement

The raw data supporting the conclusions of this article will be made available by the authors, without undue reservation.

## Author Contributions

AC developed the theoretical formalism, performed the analytic calculations, and performed the numerical simulations. CC supervised the project. All authors contributed to the final version of the manuscript.

## Conflict of Interest

The authors declare that the research was conducted in the absence of any commercial or financial relationships that could be construed as a potential conflict of interest.
